# APPLYING THE TDF FRAMEWORK TO VACCINATION DECISION-MAKING AMONG DIVERSE OLDER ADULTS

**DOI:** 10.1093/geroni/igae098.2472

**Published:** 2024-12-31

**Authors:** Taylor Patskanick, Sophia Ashebir, Alexa Balmuth, Lisa D’Ambrosio, Joseph Coughlin

**Affiliations:** Massachusetts Institute of Technology, Cambridge, Massachusetts, United States; Massachusetts Institute of Technology, Cambridge, Massachusetts, United States; Massachusetts Institute of Technology, Cambridge, Massachusetts, United States; MIT AgeLab, Cambridge, Massachusetts, United States; MIT AgeLab, Cambridge, Massachusetts, United States

## Abstract

Annual immunization rates among U.S. adults remain low relative to younger age groups and vary further by vaccine target. Gaps in research understanding the factors influencing older adults’ uptake of vaccines typically indicated for those aged 50+ such as pneumococcal, flu, tetanus, diphtheria and pertussis, shingles, and coronavirus 2019 as persist. Older adults represent a spectrum in the degree to which they may be up-to-date on these recommended vaccines and the facilitators or barriers to this uptake may further vary across vaccine targets. This paper presents findings from the qualitative portion of a cross-sectional study conducted from 2021 to 2022 in which 25 virtual focus groups and interviews of adults aged 65+ (N=112) and who identified as Black or African-American, Latine, Asian or Asian-American, or White were convened to discuss vaccination decision-making. Groups were stratified by racial/ethnic identity and degree to which participants were up to date on the aforementioned vaccinations. In this paper, data were analyzed in MAXQDA2024 using the Theoretical Domains Framework to identify and map the framework’s constructs across vaccine targets among the partially or fully vaccinated. Findings compare and contrast the prevalence of domains (e.g., social influence, emotion, knowledge) across targets among the vaccinated in our sample. Implications of this work for healthcare professionals who seek to influence health behavior change and future research focused on the implementation of interventions to improve vaccination uptake among older adults will be discussed.

